# Fostering therapeutic relationships in brief interventions: an exploratory qualitative study of the Ensemble program for informal caregivers of adults with psychiatric disorders

**DOI:** 10.3389/fpsyg.2025.1706122

**Published:** 2026-03-05

**Authors:** Hélène Wilquin, Léa Plessis, Elora Bourgade, Shyhrete Rexhaj

**Affiliations:** 1 Aix Marseille Univ, CNRS, CRPN, Marseille, France; 2 Laboratoire de Psychopathologie et Processus de Santé, Université Paris Cité, Boulogne-Billancourt, France; 3 Hôpitaux de Provence, C.H. Montperrin, HDJ Adolescents- Salon de Provence, Aix-en-Provence, France; 4 La Source, School of Nursing Sciences, HES-SO University of Applied Sciences, Lausanne, Switzerland

**Keywords:** mental health, partnership, professional-informal caregivers relations, psychosocial intervention, psychotherapeutic processes

## Abstract

**Introduction:**

Informal caregivers play a crucial and multifaceted role in the recovery of individuals with psychiatric disorders, but this role can negatively impact their own quality of life. Therefore, targeted interventions that address caregivers’ specific needs are essential. The Ensemble program is a brief, targeted intervention designed to enhance caregivers’ psychological well-being through a helping relationship centered on their lived experience. This qualitative study explored how dynamic, co-constructed therapeutic relationships develop between practitioners and informal caregivers, and how both parties experience these relationships.

**Methods:**

A qualitative design was employed, integrating semi-structured individual interviews with Ensemble practitioners and a focus group with informal caregivers. All data were audio-recorded, transcribed verbatim, and analyzed using thematic content analysis. Additional material was collected from post-program responses to the open-ended question, “What did you like most about this follow-up?”

**Results:**

Four relational dimensions characterized the informal caregiver–practitioner relationship: (1) therapeutic alliance; (2) an empathic, respectful, partnership-oriented, and accepting stance; (3) relational authenticity; and (4) flexibility, adaptation, and co-construction. A strong therapeutic relationship was shown to develop rapidly within the five-session Ensemble intervention. Facilitating factors included practitioners’ deep understanding of caregivers’ experiences, their relatives’ clinical and social backgrounds, and the challenges inherent to the caregiving role. Attentive, accurate, and non-judgmental listening promoted caregiver engagement and active participation.

**Conclusion:**

Relational reciprocity emerged as a key mechanism, supporting partnership-based dynamics and emphasizing the importance of flexible frameworks along with empathic, welcoming, and non-judgmental attitudes in fostering hope and sustained engagement.

## 1 Introduction

Across the world, informal caregivers have become essential yet often overlooked partners in the recovery process for individuals living with mental disorders, helping to provide evidence-based care in the community (Bonsack et al., 2025). Informal caregivers offer crucial support—often without formal training or institutional recognition (Lacey et al., 2024; Van et al., 2024). Their contributions are multidimensional, including practical daily assistance (such as household tasks and daily activities), emotional support during psychological distress, and help with illness and financial management (Estrada-Fernández et al., 2022). This informal support plays a key role in patients’ recovery (Petkari et al., 2021; Rodolico et al., 2022). However, providing sustained informal support can negatively impact caregivers’ own quality of life, highlighting the need for targeted interventions that address their specific needs and lived experiences (Hansen et al., 2022; Rexhaj et al., 2017a).

The Ensemble program was created by Rexhaj et al. (2017b) as a short, tailored intervention aimed at increasing caregivers’ sense of agency and enhancing their ability to manage their caregiving roles. Consisting of five one-hour sessions, its brief format aligns with informal caregivers’ expressed preference for quick and focused support. However, this brevity poses a challenge for Ensemble providers, who must build a strong therapeutic relationship within a limited period to achieve effective outcomes. The current study seeks to deepen understanding of the therapeutic relationship and its challenges in this setting.

### 1.1 Rationale and background of the therapeutic relationship

Therapeutic relationships involve multiple dimensions and are crucial across treatment settings (Lambert and Barley, 2001; Norcross, 2001; Norcross, 2002; Norcross and Lambert, 2018). They impact therapy’s course and effectiveness, facilitating change to reduce suffering and improve well-being. Research shows therapeutic change relates more to common factors than specific techniques (Iversen et al., 2025; Keefe et al., 2020; Lambert and Barley, 2001). The process is complex, requiring examination of both technique-specific and shared factors, with the therapeutic relationship playing a key role (Finsrud et al., 2022). This relationship involves understanding patients’ life contexts, respecting commitments, and genuine interest in their experiences and goals (Brewer and Giommi, 2025; Iversen et al., 2025; Keefe et al., 2020) Furthermore, each client–therapist relationship is unique, and its intersubjective nature creates methodological challenges in identifying and measuring influencing factors (Eilertsen and Eilertsen, 2023). The definition of a therapeutic relationship itself varies (Horvath and Bedi, 2002), with Gelso and Carter describing it as the set of “all of the feelings, attitudes, and behaviors, conscious and unconscious, occurring between two people, where one is a professionally sanctioned help giver and the other a client, patient, or the like” (Gelso and Carter, 1985, p. 159). Their definition highlights the evolving and intersubjective nature of the relationship. It extends beyond the traditional provider–patient framework to encompass other helping relationships. The therapeutic relationship can also be characterized by its components, which encompass alliance, empathy, positive regard/acceptance, curiosity, authenticity, or collaboration/partnership (Brewer and Giommi, 2025; Norcross and Wampold, 2011), and which are often studied separately. Among these, therapeutic alliance remains the most studied. Therapeutic alliances are widely recognized as reliable predictors of treatment outcomes (Horvath et al., 2011; Horvath and Bedi, 2002; Manubens et al., 2023). It can be studied from the very start of therapy and is decisive for the course of therapy (Del Río Olvera et al., 2022; Flückiger et al., 2020). A meta-analysis of 295 studies reported an average correlation of *r* = 0.28 between alliance quality and treatment outcomes (Flückiger et al., 2018).

The therapeutic relationship and its components have rarely been explored deeply, often studied only at specific moments like the start of therapy rather than as a dynamic process (Marchi et al., 2024). Similarly, in informal caregiving, research mainly focuses on measurable outcomes like symptom reduction or caregiver satisfaction, with limited attention to the quality and development of the therapeutic relationship itself (Petkari et al., 2021; Rexhaj et al., 2023; Rodolico et al., 2022; Van et al., 2024).

However, understanding the relational aspect is essential. The therapeutic relationship helps practitioners understand the specific experiences of informal caregivers, including their challenges, emotional burdens, and lived realities that are often unnoticed. Building a strong, collaborative alliance can improve informal caregivers’ sense of agency, support their emotional skills, and prevent the risk of burnout. Addressing this gap is vital to capturing the complexity of the therapeutic relationship involving informal caregivers and to fostering a more integrated approach, as demonstrated by the Ensemble program (Rexhaj et al., 2023). This reasoning emphasizes the importance of studying therapeutic relationships not just from the perspective of traditional therapy with patients but also in brief, empowerment-based interventions for informal caregivers. The current study aims to contribute to a deeper understanding of how therapeutic relationships support informal caregivers effectively in the Ensemble context.

### 1.2 The Ensemble program: a brief intervention for informal caregivers of individuals with psychiatric disorders

The Ensemble program is a short five-session intervention aimed at supporting informal caregivers of individuals with psychiatric disorders, promoting their well-being, and helping them maintain a caregiving/work/life balance (Rexhaj et al., 2023). Although Ensemble’s relational framework differs from patient-centered psychotherapy—usually involving treatment delivery—it remains rooted in a supportive helping relationship. The program aims to enhance informal caregivers’ psychological well-being by activating their capacity for action within their own contexts (Rexhaj, 2019). Developed in collaboration with informal caregivers, Ensemble addresses specific support needs regardless of the care recipient’s diagnosis.

Informal caregivers often face judgment, stigma, and social isolation, which can prevent recognition of their role and acknowledgment of their challenges, highlighting the need for support that encourages reflection on their lived experiences (Kaas et al., 2003; Magaña et al., 2007). Informal caregivers are crucial to the mental health care system, offering vital daily support to individuals with psychiatric disorders (Drapalski et al., 2008). However, research has shown that this role can significantly impact the mental health and quality of life of informal caregivers (Bauer et al., 2012; Radfar et al., 2014; van der Voort et al., 2009).

### 1.3 Practitioner–informal caregiver relationships within the Ensemble program

The Ensemble program is built on intersubjective encounters between practitioners and informal caregivers across five sessions, where the relationship quality is central in co-constructing tailored proposals and rapidly implementing individualized, effective support strategies. The first session welcomes the informal caregiver by providing a safe and supportive setting that encourages them to share their lived caregiving experiences (Rexhaj et al., 2017b). This session is crucial for fostering engagement for both practitioners and informal caregivers.

Informal caregivers often do not anticipate healthcare services or therapeutic interventions, viewing such support as part of their ill relatives. Moreover, practitioners are accustomed to working with patients in long-term care or emergency situations. Many healthcare professionals remain unfamiliar with interventions aimed at family caregivers, whose experiences are frequently misunderstood, stigmatized, and socially isolated (Drapalski et al., 2008; Magaña et al., 2007). Reciprocal engagement in the program is crucial from the very first encounter, as it enables sharing and acknowledgment of experiential knowledge regarding the informal caregiver role and the recovery process, regardless of the therapeutic path of the relative living with a disorder (Fiest et al., 2018; Jørgensen et al., 2025).

The informal caregiver and practitioner can co-construct targeted and adaptive interventions through attentive and intersubjective listening and the interplay of interactions during these early meetings. Rather than imposing predefined outcomes, this process supports caregivers in recognizing and mobilizing their capacity for action in specific situations.

To support informal caregivers during the intervention, practitioners receive two-and-a-half days of specialized training designed to integrate the key aspects of caregiving and equip them with support tools. The training comprises four modules: (1) Understanding informal caregivers’ lived experiences and the principles of health education; (2) detecting informal caregiver distress and identifying needs, difficulties, painful emotions, and social resources using clinical tools; (3) acquiring support techniques, including emotion management, problem solving, communication, and recognizing of personal values and resources; (4) understanding the dynamics of the therapeutic relationship and developing a professional perspective around the informal caregiver’s experience (Rexhaj et al., 2023).

The final module included role-playing exercises to help practitioners consider the informal caregiver’s unique journey, shift focus away from the patient’s illness, cultivate curiosity and presence, and view Ensemble as a co-constructed intervention rather than a prescriptive approach (Iversen et al., 2025; Keefe et al., 2020). Subsequently, clinical supervision helps refine the practitioner’s professional outlook and improve skill development through real-world practice (Nguyen et al., 2021).

Therapeutic relationships were the main focus for practitioners within the Ensemble program. In their training, three key components are emphasized to enhance the brief intervention process: agreement on objectives for a dyadic collaboration, quality of the relational bond based on mutual trust, and informed reciprocal engagement (Bordin, 1979; Keefe et al., 2020).

#### 1.3.1 Agreement on objectives: a dyadic collaboration

Within Ensemble, the informal caregiver is viewed as a partner instead of a passive care recipient. The therapeutic process is seen as a co-construction based on trust and collaboration, where the informal caregiver is as actively involved as the practitioner. While the practitioner offers professional expertise, the informal caregiver provides valuable experiential knowledge, and both are essential to the intervention (Dumez and L’Espérance, 2024). Support activities are customized to meet the caregiver’s needs and challenges, and are influenced by the intersubjective dynamic that develops during partner interactions (Rexhaj et al., 2017b). This partnership perspective, which emphasizes the notion of being “allies,” is central to the Ensemble program.

It also echoes the collaborative dimension of the therapeutic alliance as conceptualized by Bordin (1979), which is rooted in the notion of the “working alliance” theorized by Greenson (1965) in psychoanalysis. The therapeutic alliance reflects the necessary cooperation between the client and therapist, sustained by the client’s trust in the practitioner and a reciprocal, consistent commitment to the process.

A strong alliance requires agreement and clarity about the objectives and the means used to achieve them, which is supported by the practitioner’s collaborative style to create a dyadic construct (Wampold and Flückiger, 2023). Therefore, establishing a clear dyadic collaboration is an integral component of this partnership, representing a key principle of the Ensemble program.

#### 1.3.2 Quality of the informal caregiver–practitioner relationship

Ensemble practitioners were encouraged to adopt an empathic, respectful, and open attitude throughout the intervention. This perspective aligns with empathic and unconditional positive regard. Rogers (1970) defined the state of empathy as “perceive the internal frame of reference of another with accuracy and with the emotional components and meanings which pertain thereto as if one were the person, but without ever losing the ‘as if’ condition.” Thus, empathic understanding refers to the practitioner’s ability to comprehend the informal caregiver’s lived experience without merging with it. This capacity is a core element of the therapeutic relationship, facilitating genuine contact with the client’s worldview and contributing significantly to therapeutic outcomes (Norcross and Lambert, 2018).

Unconditional positive regard entails nonjudgment, acceptance, and a consistently supportive orientation (Iversen et al., 2025). It involves welcoming the client, recognizing their strengths and vulnerabilities, and affirming their potential for change. This approach requires warmth and kindness, with many studies showing its positive impact on therapeutic effectiveness (Norcross and Lambert, 2018).

#### 1.3.3 Reciprocal engagement of the informal caregiver–practitioner

Congruence or authenticity is central to the therapeutic relationships (Norcross and Lambert, 2018; Rogers, 1970). Ensemble practitioners are encouraged to combine a solid framework with flexibility and adaptability, customizing support to meet the individual needs of informal caregivers. This flexibility applies both to the tools and strategies used and to practical aspects such as the setting or session schedule.

In psychotherapy research, adaptability, often referred to as responsiveness, is linked to better outcomes, as therapists who adjust to clients’ needs, defenses, and feedback are seen as more effective (Norcross and Wampold, 2011; Wu and Levitt, 2020). Therefore, flexibility and reciprocal engagement are key to maintaining a strong therapeutic alliance in brief interventions such as Ensemble.

### 1.4 Study aim

This study examined how dynamic, co-constructed therapeutic relationships develop between practitioners and informal caregivers within the brief, empowerment-focused Ensemble program, aiming to optimize support tailored to the lived experiences of informal caregivers. Two specific research questions guided this investigation: (1) How do practitioners describe their therapeutic relationships in relation to informal caregivers’ satisfaction—or dissatisfaction—with the Ensemble program? (2) How do informal caregivers perceive and experience their relationships with Ensemble practitioners throughout the intervention?

## 2 Materials and methods

### 2.1 Context of the study: from evaluation of the Ensemble program to the role of the practitioners- informal caregivers relations

#### 2.1.1 Evaluating the effectiveness of the Ensemble program in the French context

The participants in this study were part of a larger research project. This broader project, which combines quantitative and qualitative methods, aimed to evaluate the effectiveness and adaptability of an Ensemble program in the French context.

Quantitative data were collected through a randomized controlled trial. Eighteen adult family caregivers of individuals with severe mental disorders were randomly assigned to either the Ensemble group (Gexp), which received the intervention (*N* = 9), or the support as usual group, which did not receive the intervention (*N* = 9). Multiple self- reported measures (e.g., caregiver burden, optimism level, psychological health, and quality of life) were collected at two time points: pre-test (T0, before randomization) and post-test (T1, two months after T0), which corresponded with the end of the Ensemble program. Results showed that the psychological health and optimism levels of informal caregivers improved after the Ensemble program, whereas there was no improvement for those in the support as usual group (Wilquin et al., 2020).

In this broader study, qualitative data were also collected: a focus group was used to gather personal experiences and identify barriers and facilitators conducive to the changes aimed for with the Ensemble program. This qualitative approach allowed participants in the Ensemble group (Gexp) to share and reflect on their experience participating in the Ensemble program. In total, four volunteer informal caregivers participated in this focus group 5 months after completing the brief intervention. Within this qualitative methodology, Gexp participants answered open-ended questions about their satisfaction with the program.

#### 2.1.2 Exploration of the dynamic, co-constructed therapeutic relationships develop between practitioners, and informal caregivers within the Ensemble program

The Ensemble program offered a unique environment to examine therapeutic relationships within the context of brief, structured support for informal caregivers. Specifically, this study aims to explore the dynamics of these relationships, including interpersonal processes that influence therapeutic engagement. Therefore, an approach that captures intersubjectivity—the shared meanings and mutual influences between informal caregivers and Ensemble practitioners is needed.

To explore this more specific issue, two qualitative data sets were examined and compared: one focusing on the experiences of Ensemble practitioners and the other on the experiences of informal caregivers who had benefited from the program. For that, a specific design was adopted for the present study: (1) First, individual research interviews were specifically conducted with Ensemble practitioners to explore the therapeutic relationship experienced professionally, (2) In the second step, using a deductive approach, data previously collected as part of the larger study from the focus group conducted with informal caregivers of the Gexp were reanalyzed to select and examine only portions of the corpus related to the experience of the therapeutic relationship of informal caregivers. Additionally, the responses provided by Gexp participants to the open-ended question, ‘Can you tell us what you liked best about this follow-up?’ in the broader study were also explored, aligning with the more specific research question of this study.

Both these qualitative methods (interviews and focus group) are detailed in the following sections (design, conduct, data analysis, and findings) according to the consolidated criteria for reporting qualitative research (COREQ checklist) (Tong et al., 2007). A thematic analysis method had been used to identify emerging themes in participants’ discourse (Braun and Clarke, 2006, 2019).

### 2.2 Participants

#### 2.2.1 Recruitment of informal caregivers

Informal caregivers participating in the broader study were recruited from the Union nationale de familles et amis de personnes malades et/ou handicapées psychiques (UNAFAM) and the Schizophrenia Expert Center of the Aix-Marseille region. The inclusion criteria were: (a) having an adult relative with a psychiatric disorder (with or without a formal diagnosis); (b) 18 years of age or older; (c) residing in or near the Aix-Marseille region; (d) proficiency in French; and (e) a burden score greater than 20 on the 22-item Zarit Burden Interview (Piette, 2015). Anyone interested in the study was offered a personalized telephone interview to clarify the requirements for participation. Gexp participants were invited to a focus group to share their experiences, needs, and expectations (Krueger and Casey, 2015). The focus group allowed them to meet and discuss their experiences participating in the Ensemble program.

#### 2.2.2 Recruitment of Ensemble practitioners

Two psychologists with PhDs, trained to deliver the Ensemble program, administered it to the nine Gexp participants in the larger study. Each participant was randomly assigned to one of these two practitioners, whom they had never met before. Once the research program for the larger study was completed, each of these two practitioners was asked to reflect on a therapeutic relationship developed during the program with a particular informal caregiver. The researchers specified to each practitioner which therapeutic relationship they were asked to comment on: two informal caregivers who reported contrasting levels of satisfaction with the Ensemble program and the research environment (a satisfaction score of 20/28 versus a satisfaction score of 28/28) were chosen.

### 2.3 Qualitative data collection

Two qualitative data collection methods were employed: individual semi-structured interviews conducted with Ensemble practitioners and a focus group with Ensemble informal caregivers (Kallio et al., 2016). These complementary approaches enabled the exploration of both practitioners’ and informal caregivers’ experiences within the program.

#### 2.3.1 Individual semi-directive interview

A research assistant trained in clinical psychology, with only brief, theoretical knowledge of the Ensemble program, conducted individual telephonic interviews with each of the two Ensemble practitioners. The three individuals involved (research assistants and practitioners) knew each other professionally and had already met in person previously. Both interviews were conducted and audio-recorded while the research assistant and practitioner were alone in a quiet room, ensuring confidentiality. Each interview lasted approximately one hour and took place 10 months after the practitioner had completed delivering the Ensemble program to the informal caregiver, focusing on the therapeutic relationship. Individual interviews enabled the collection of subjective information about a person’s experiences, promoting personal expression and exploring specific themes (Fernandez and Pedinielli, 2006). A semi-structured interview guide was prepared to facilitate the interviews and was designed to address the therapeutic relationship experienced professionally by Ensemble practitioners (Supplementary material 1).

The first section of the guide included open-ended questions exploring the Ensemble practitioner’s overall perception of the therapeutic relationship with the informal caregiver. Questions included, “How did you experience the relationship between yourself and the informal caregiver you supported during the five sessions of the Ensemble program? Can you describe this relationship?” and “In your view, what were the most important or striking aspects of the relationship with the informal caregiver?” Additional questions in the guide aimed to: (1) explore the quality of the therapeutic alliance; (2) explore the practitioner’s subjective experience of the relationship; and (3) examine the significance of the practitioner’s characteristics and attitudes, including empathy, unconditional acceptance, absence of judgment, authenticity, ability to manage relational ruptures, capacity to provide secure attachment and manage emotional engagement, and adaptability to the caregiver’s individual characteristics. The interview concluded with the following question: “Is there any aspect of your relationship with the informal caregiver you have not yet discussed that you would like to mention before we close the interview?”

#### 2.3.2 Focus group interview

The four Gexp participants who agreed to take part in the focus group met at Aix-Marseille University in a private and comfortable behavioral research room (H2C2 technological platform). All discussions were audio recorded. A research assistant, clinical psychologist, and expert in conducting research interviews (PhD in psychology), who was unfamiliar with the Ensemble program, facilitated the session, ensuring active listening, respectful turn-taking, and a supportive atmosphere. This assistant had never met or known the life stories of the focus group participants before this group interview. The focus group facilitator was to ensure adherence to the method, which includes promoting interaction among participants; knowledge sharing; idea generation; and exploring human, relational, affective, and emotional dimensions while encouraging the sharing of individual perspectives (Krueger and Casey, 2015).

A semi-structured interview guide was developed to direct the discussion toward the participants’ experiences with the Ensemble program (Supplementary material 2). The introductory questions addressed the research context, focus group objectives, facilitator role, session duration (approximately 2 hours), and confidentiality procedures. Core questions explored the program’s added value, processes of change, and suggestions for improvement, focusing on the intersubjective caregiver–practitioner relationship. These questions were: “Could you describe a particularly significant situation you experienced during the Ensemble program?”; “What helped you cope with this situation?”; and “Is there anything you wish had been provided more, or anything you felt was insufficient?” This approach facilitated the sharing of individual perspectives while exploring the program’s relational, emotional, and experiential dimensions.

#### 2.3.3 Specific qualitative questions about the quality of the intervention

Two months after completing the brief and targeted Ensemble intervention, the informal caregivers were invited to complete and return the questionnaires by mail. The questionnaire focused on the participants’ satisfaction with the program, and data collection procedures were designed to ensure anonymity. The open-ended question asked, “Can you tell us what you liked best about this follow-up?”

### 2.4 Data analyses

This study applied thematic content analysis to a complete set of verbatim transcripts. The two datasets—the transcripts produced by the Ensemble practitioners during individual telephone interviews and those produced by informal caregivers during the focus group—were analyzed separately. An inductive approach was first employed as follows: (1) listening to the audio recordings multiple times to immerse the researchers in the participants’ subjective experiences; (2) word for word transcription of the recordings; (3) identifying recurring elements in the transcripts, such as repetition of words or ideas, use of specific semantic fields, and emotional tone of the discourse; and (4) categorization of statements sharing common meaning into discourse themes (Castillo et al., 2021). The themes from the thematic content analysis of the practitioners’ discourse were then used in a data-driven coding approach to analyze the discourse of informal caregivers regarding the therapeutic relationship.

As the focus group addressed the overall experience of caregivers participating in the Ensemble program, only portions of the corpus referring to the experience of the therapeutic relationship were selected for analysis in the present study. Of the 575 meaning units comprising the entire focus group transcript, 78 (13.5% of the initial corpus) were retained. These 78 units were annotated using a deductive method, and each unit was assigned one of the therapeutic relationship themes identified from the thematic analysis of the two Ensemble practitioner interviews. Double-coding was applied to these 78 units to generate discussions within the research team. These discussions gradually refined the assignment of themes to informal caregivers’ statements on therapeutic relationships. Inter-annotator agreement was calculated using Cohen’s Kappa coefficient, indicating a good level of agreement (*κ* = 0.745; *z* = 8.87; *p* < 0.001) (Halpin, 2024).

### 2.5 Ethics

Participants were informed about the study and their rights and sign a written informed consent form. All necessary measures were taken to ensure confidentiality and data protection in line with the standards of the Ensemble randomized controlled trial, as originally described in the protocol by Rexhaj et al. (2020) and conducted in Switzerland. Specifically, researchers were especially careful to respect and consider the well-being and integrity of participants during the focus group interviews. Before the interview began, oral information was repeated, and participants were informed about confidentiality, anonymity in data presentation, and their right to withdraw at any time. All audio recordings and transcripts were securely stored on the platform used by Aix Marseille University, which provides a safe way to archive and preserve the data long-term. Access to these data files is limited to the research team. In fact, fully anonymizing all research data (i.e., removing all personally identifiable information) is challenging, and sharing it would violate the GDPR (General Data Protection Regulation). Furthermore, participation in this study was voluntary, and no incentives or compensation were offered. To ensure equal access, participants facing financial difficulties could be reimbursed for transportation costs related to attending the interviews. Only one participant was in need.

## 3 Results

### 3.1 Sociodemographic data of informal caregivers in the Ensemble program

Nine family caregivers with a mean age of 53.5 years (SD = 11.6) were part of the Gexp. Most caregivers were parents (75%), predominantly women (*n* = 8; *n* = 1 man), and most supported male relatives (*n* = 7; n = 2 women). The majority were employed (*n* = 5), and three were retired. The participants’ mean satisfaction score regarding the Ensemble program was 21.15 ± 3.44. The care recipients had been living with schizophrenia (*n* = 6) or bipolar disorder (*n* = 3) for an average of 15.1 years (SD = 13.9). Their ages ranged from 20 to 69 years. The frequency of contact between caregivers and their relatives varied from daily to monthly, depending on the family situation. Detailed participant characteristics are presented in Table 1.

**Table 1:** Sociodemographic data of Ensemble informal caregivers (*N* = 9).

Participant number	Informal caregiver sex	Age (in years)	Socio-professional category	Relationship with the afflicted relative	Duration of the illness of the relative (in years)	Mental disorder (if diagnosed)	Relative gender	Age of the relative (in years)	Frequency of contact with the relative suffering from a mental disorder
1^a^	M	46	Employee	Father	1	Schizophrenia	M	20	Daily
3^b^	F	40	Employee	Daughter	41	Bipolar disorder	F	NA	Weekly
4^a^	F	66	Employee	Mother	24	Bipolar disorder	F	40	Daily
8^a^	F	50	Employee	Mother	5	Schizophrenia	M	23	Daily
9	F	69	Retired	Mother	25	Schizophrenia	M	43	Monthly
10^b^	F	64	Retired	Mother and daughter	20 and 30	Schizophrenia	M	40 (son) and 69 (brother)	Daily for his son and no contact for 6 months for his brother
11	F	61	Retired	Mother	4	Bipolar disorder	M	30	Weekly
18	F	37	Employee	Ex-partner	12	Schizophrenia	M	39	Weekly
17^a^	F	48	Employee	Mother	1	Schizophrenia	M	20	Daily

a Informal caregivers participating in the focus group, for whom the therapeutic relationship was explored from their own perspective as beneficiaries of the program.

b Informal caregivers whose therapeutic relationships were explored from the perspective of the Ensemble practitioner.

M, Male; F, Female.

Participants 3 and 10 from the Gexp who rated the Ensemble program and research conditions as moderately and highly satisfactory, respectively, were selected to explore the therapeutic relationship from the perspective of Ensemble facilitators.

Participant 3, a 41-year-old informal caregiver supporting her mother—diagnosed with schizophrenia—had previously attended support groups but reported not feeling listened to or recognized in her caregiving role. From the first session of the Ensemble program, she expressed a need to reclaim her identity (differentiated from that of her mother) and draw upon her positive and resourceful professional identity. Participant 3 had a Zarit score of 58 prior to starting the program and a score of 52 upon completion of the Ensemble intervention. At the end of the program, Participant 3’s satisfaction score for the Ensemble intervention was 20.

Participant 10, aged average 64, was an informal caregiver for both her son and brother, each diagnosed with schizophrenia. She had extensive knowledge of the associative sector and benefited from individual support outside the program. From the beginning of the Ensemble sessions, she expressed a need for listening, recognition, and perspective on a family environment marked by multiple difficulties, including legal issues. Participant 10 had a Zarit score of 60 before starting the program and a score of 3 after completing the Ensemble intervention. At the end of the program, Participant 10’s satisfaction score for the Ensemble intervention was 28.

Participants 1, 4, 8, and 17 from the Gexp participated in the focus group.

Participant 4, aged 66 years, was the informal caregiver of her 40-year-old daughter, who had been living with bipolar disorder for several years. She has been involved in support groups organized by family associations for approximately 20 years. Participant 4 had a Zarit score of 21 before starting the program and a score of 16 after completing the Ensemble intervention. At the end of the program, Participant 4’s satisfaction score for the Ensemble intervention was 27.

Participant 8, aged 50 years, was the informal caregiver of her 24-year-old son, who had experienced psychotic symptoms for several years, although his diagnosis remained uncertain. She had received individual support from a psychiatrist and was engaged in a family caregiver association. Participant 8 had a Zarit score of 27 before entering the program and a score of 23 after completing the Ensemble intervention. At the end of the program, Participant 8’s satisfaction score for the Ensemble intervention was 26.

Participants 17 and 1 were the parents of a 20-year-old son diagnosed with schizophrenia, whose first psychotic episode and hospitalization occurred approximately one year prior. For this couple, the Ensemble program represented their first experience with a structured support intervention. Participant 17 had a Zarit score of 42 at baseline and a score of 24 after completing the Ensemble intervention. Participant 1 had a Zarit score of 45 prior to the program and a score of 8 following the same intervention. At the end of the program, Participant 17’s satisfaction score for the Ensemble intervention was 28. The satisfaction questionnaire was not completed by Participant 1, so no score was therefore available.

### 3.2 Practitioner thematic analysis results on the therapeutic relationship

The practitioners’ perspectives allowed the identification of four therapeutic relationship themes under two conditions: (a) a caregiver who rated the Ensemble intervention as satisfactory, and (b) a caregiver who rated the Ensemble intervention as less satisfactory. These four themes were (1) therapeutic alliance; (2) empathic, respectful, partnership-based, and accepting stance; (3) authenticity within the relationship; and (4) flexibility, adaptation, and co-construction.

The themes were defined as follows. “Therapeutic alliance” refers to aspects of collaboration within a relationship. It also encompasses the discourse related to agreement (or disagreement) between the practitioner and the caregiver regarding the intervention objectives. This theme includes the responsibilities of each party.

The “empathic, respectful, partnership-based, and accepting stance” theme pertains to empathic understanding in relationships. This reflects the practitioners’ positive regard for the caregiver, acknowledging their perspectives and values. It also encompassed the recognition of the caregiver’s potential for change throughout the five sessions. This relationship is based on the principle of mutual recognition.

“Authenticity within the relationship” captures the caregiver’s or practitioner’s attentiveness to their own experiences within the relationship. This involves active engagement from both parties and includes maintaining the professional dimensions of the relationship.

The “flexibility, adaptation, and co-construction” theme reflects the capacity to co-construct the support process, considering the specificities of both caregivers and practitioners, including experiential and professional knowledge. It also encompassed the ability to maintain relationships despite occasional tensions or disagreements during the intervention.

Verbatim excerpts illustrating these four themes are presented in Table 2. Across both conditions, differences emerged in how practitioners described each theme. When the intervention was seen as satisfactory (informal caregiver 10), practitioner A emphasized an empathetic stance, flexibility, and co-construction, highlighting fluid collaboration and shared engagement. In less satisfactory cases (informal caregiver 3), practitioner B noted challenges in the therapeutic alliance and goal alignment, highlighting the need for flexibility to meet caregivers’ expectations. Some saw the aim of being non-judgmental as sometimes leading to broad permissiveness, causing ambiguity about guidance levels. Regarding authenticity, practitioners sometimes felt uncomfortable sharing their experiential stance, limiting transparent responses. Overall, engagement with these four themes varied based on the perceived relationship quality climate.

**Table 2:** Practitioners’ perspective on the therapeutic relationship in Ensemble based on the satisfaction of informal caregivers and on the four themes of the therapeutic relationship.

Four themes of the therapeutic relationship	Relationship of Practitioner A with Participant 10	Relationship of Practitioner B with Participant 3
	Very satisfied with the program and the study	Moderately satisfied with the program and the study
	Verbatim	
Therapeutic alliance	“From the beginning, it was clearly stated that it’s five sessions… [Yes, you really set the framework, the objectives, all that, did you discuss it together? In the end, was it clearly said? Clearly exchanged?] Yes, absolutely.” “I was still able to tell her that I remained available, in case there was any question.” “Even if she was always willing, etc., there were times when I could propose an appointment, and, for example, it was during her aquagym hour or something she valued. She very clearly told me: ‘Oh no, I care about that, can we reschedule? It’s my only session.’”	“Her request was more in the realm of supportive therapy rather than the Ensemble program itself.” “I think she may have expected a lot from this program, but perhaps I did not present it to her as I should have initially, so in fact, we were not on the same path; we had taken two different routes, parallel but still different.” “It’s not a clear success, and I think this is because I may not have clearly stated the objectives of this program from the start.”
Empathic, respectful, partnership-based, and accepting stance	“A very benevolent attitude, letting her choose the table where we would sit, offering her what she wanted to drink, playing, so to speak, the role of a server… Not interrupting her too much and letting her express what she wanted to share.” “Even if I felt boredom sometimes, I thought: yes, at the same time, you are not doing this for yourself… This lady needs to vent, to repeat things several times… in acceptance.”	“I think that at times, it triggered reactions that were perhaps outside the psychotherapeutic framework, in the sense that, well, sometimes I thought: it’s all well and good, but it’s a bit easy to judge. She does not have children and allows herself to say that others are poorly raised, sometimes using very aggressive language. Through this, I thought she expects me to react to it.” “I felt that there was perhaps some distancing in the relationship, because I tried to make her understand that this… I would say this hatred, maybe too strong, but this disapproval of others was only harming herself, her way of perceiving herself and the world. She only hurts herself, because people have the right to be different.”
Authenticity within the relationship	“It’s still very, very authentic.” “I heard someone call me, and I turned around, and it was someone from my surroundings… I refocused on the interview… This is an example of someone who called me, and I did not even allow myself to go see them… I must not deviate from my mission.”	“At times, it still triggered reactions that were perhaps outside the psychotherapeutic framework… Maybe I should have reacted, but I preferred not to say much.” “I wasn’t necessarily very comfortable.”
Flexibility, adaptation, and co-construction	“Having a good understanding of what mental illness is, I think, helps a lot in supporting these people, by not saying things that would be incongruous given the situations they have experienced.” “When I brought her during the inter-session to appropriate tools or things I had presented… when she took the tools, it was to use them differently. So it was somewhat surprising and interesting at the same time… there was always material to discuss, to elaborate.”	“This first interview was quite complicated, and afterwards… well, it was certainly a form of self-protection on my part, because I did not manage to structure the first meeting well, so perhaps in the following meetings, I may have structured it a little too much, where it wasn’t necessarily her request.” “I had told her: it would be interesting for you to write what you experienced… she said: ‘Yes, but if I’m not read, I do not really see the point of writing.’ … She said: ‘Yes, but I’m tired of always talking about the positive when in fact it’s negative.’ Well, it’s okay, write anyway… and in the end, she never returned to it.”

These four dimensions of the therapeutic relationship, as identified by practitioners, were then used in a data-driven coding approach to analyze how informal caregivers experience and interpret the relationship, thereby connecting professional perspectives with lived experiences of participants.

### 3.3 Informal caregivers’ thematic analyses and results of the therapeutic relationship

**Figure 1:**
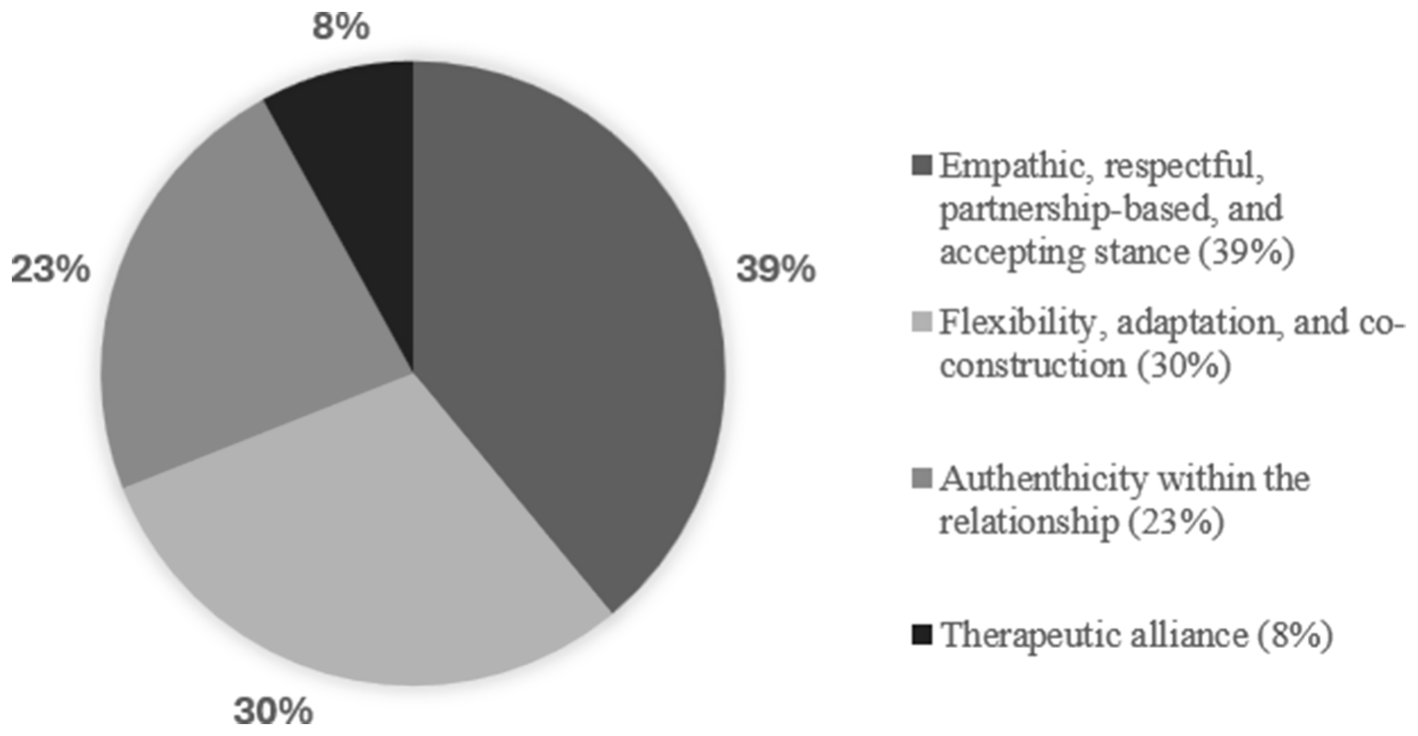
Percentage distribution of the four therapeutic relationship themes presented in the Ensemble beneficiaries’ discourse.

The analysis of how often meaning units related to the therapeutic relationship were spontaneously mentioned during the focus group exploring participants’ overall experience of the Ensemble program helped us evaluate the importance of each theme in informal caregivers’ lived experiences. Empathic, respectful, partnership-based, and accepting stances emerged as the most frequently cited theme (39%), followed by flexibility, adaptation, co-construction (30%), authenticity within the relationship (23%), and therapeutic alliance (8%) (Figure 1).

#### 3.3.1 Empathic, respectful, partnership-based, and accepting stance

This theme, which informal caregivers most frequently mentioned, highlights the importance of adopting an empathic and respectful stance to foster a sense of partnership and acceptance. All four participants explicitly mentioned this theme, indicating that it was consistently recognized across the focus group. Neutrality and attentive listening are essential for creating a climate of trust, promoting a sense of hope among caregivers, and recognizing the potential for change.

Participant 8: “What I also appreciated, and what should be considered for the program, is what you said—a neutral stance that helps us to open up as well. And within the program, there is this practical exercise, something we want to do but don’t dare, and there we feel indirectly supported, without judgment. I find this very important, and this neutrality allows us to go for it—at least that was my experience.”

#### 3.3.2 Flexibility, adaptation, and co-construction

The second theme highlights the importance of practitioners’ flexibility and ability to adapt to informal caregivers’ specific needs and temporality. Three of the four participants explicitly mentioned this theme, suggesting that it was shared by the majority of the focus group. Availability and co-construction were perceived as concrete facilitators of change.

Participant 1: “So she suggested doing something, because in talking, she pointed out that I was no longer really going out, moving around, and so on. And because she put it into words, I started to process it in my head, gradually working on it little by little, and when I saw her the next time, I told her: Yes, indeed, this is something I need to work on, and it’s taking shape.”

#### 3.3.3 Authenticity within the relationship

The third theme emphasizes the value of authenticity within the relationship. All four participants clearly mentioned this theme, showing it was consistently acknowledged throughout the focus group. Informal caregivers recognized the uniqueness of each practitioner’s style and the sincerity of their engagement, while also expressing their authenticity in the interactions. Reciprocal authenticity contributes to a relationship that is perceived as genuinely intersubjective.

Participant 4: “What I don’t know is whether it’s the program or L [Practitioner B]’s personality that made the difference.”

#### 3.3.4 Therapeutic alliance

Finally, the therapeutic alliance theme was much less frequent in participant discourse. Informal caregivers share agreement on the intervention objectives. Three of the four participants explicitly mentioned this theme, suggesting that it was shared by the majority of the focus group. This alliance facilitates decision-making and promotes the emergence of new perspectives.

Participant 8: “This really opened my mind a lot, and if I had to summarize it myself, it allowed me to refocus on myself. It really helped me… ‘don’t scatter yourself, it’s good to care for your son, but also think of yourself,’ and I think I wasn’t doing that before.”

These analyses help identify the dimensions most valued by informal caregivers and show how they directly improve the quality of the therapeutic relationship by promoting attentive listening, recognition, and a safe space for reflection and action.

Taken together, the informal caregivers’ accounts confirmed three of the four themes as key components of their relational experience (empathic stance; flexibility; authenticity). In contrast, the therapeutic alliance seemed less prominent. This pattern partially reflects the practitioners’ thematic structure but suggests that informal caregivers prioritize relational attitudes and interpersonal presence over collaborative goal-setting. This difference in thematic prominence provides further insight into how each group perceives the same relational process.

### 3.4 The quality of the therapeutic relationship: the aspect most valued by informal caregivers

Participants valued the quality of the therapeutic intervention, emphasizing specific characteristics, such as becoming more emotionally aware and the opportunity for access to a professional space for self-reflection in a secure and targeted way. The responses are shown in Table 3.

**Table 3:** Perceived benefits of the Ensemble follow-up: caregiver reflections on the therapeutic relationship.

Can you tell us what you liked best about this follow-up?	
Themes	Text participants’ responses
Self-reflection and emotional awareness	“Opportunity to focus on myself, my feelings, and my emotions. A more suitable listening regarding my ill relative.”
Quality of the therapeutic relationship	“Quality of the relationship (availability, kindness, non-judgment).” “Attentive listening of the provider.”
Professionalism and tailored support	“Listening and also the tools provided (exercises, active personal work…).” “Listening, advice, and professionalism.” “Attentiveness and intelligence of my interlocutor. Finesse of her interventions.”
Feeling supported and less isolated	“Feeling less alone when facing the difficulties.” “No judgment on my experience and strong moral support, guidance ++ towards options to ‘reflect upon’ to make personal choices.”

## 4 Discussion

This qualitative study is part of a project evaluating the feasibility and adaptation of the Ensemble intervention in the French context, which offers a suitable framework for exploring therapeutic relationships within brief, structured support for informal caregivers. Two complementary data sources were used to support this goal: (1) individual interviews with two practitioners and (2) a focus group with four informal caregivers. Overall, the findings confirm the relevance of the core dimensions of the therapeutic relationship commonly documented in the literature (Brewer and Giommi, 2025; Iversen et al., 2025; Bordin, 1979). These include the therapeutic alliance; an empathic, respectful, accepting, and partnership-oriented attitude; authenticity within the relationship; and flexibility, adaptation, and co-construction. Most importantly, the study provides new insights into how these dimensions are expressed within an intervention focused on informal caregivers. The findings indicated that these dimensions were clearly articulated by Ensemble practitioners, who were directly asked to reflect on their therapeutic relationships with informal caregivers. More notably, the thematic analysis, supported by good inter-rater reliability, revealed that these four dimensions of the therapeutic relationship were spontaneously expressed by informal caregivers during the focus group, even though the discussion guide did not explicitly aim to explore them. The focus group was instead designed to explore their broader experience of the intervention, which highlights the implicit importance of the therapeutic relationship dimensions.

These results suggest two main observations: (1) The therapeutic relationship dimensions identified in this study between informal caregivers of individuals with mental disorders and practitioners of the Ensemble program were consistent with those documented in the literature on patient–clinician relationships. (2) The identification of a reciprocal relational dynamic, with shared aspects of the therapeutic experience described by both practitioners and informal caregivers, supports the view that the therapeutic relationship is co-constructed and mutually perceived rather than defined in a unidirectional manner.

The current findings also highlight two main dimensions in caregivers’ reports: (1) an empathic, respectful, accepting, and partnership-focused attitude, and (2) flexibility, adaptability, and co-construction, which together make up nearly 70% of the spontaneously shared content related to their experience of the therapeutic relationship within the Ensemble program. In essence, acknowledging caregivers’ experiential knowledge through an empathic and non-judgmental approach, while offering tailored, real-time support, seems to be among the most meaningful—and ultimately most helpful—aspects of the relationship built through the Ensemble program. These results are consistent with a meta-analysis of 53 studies showing that the therapeutic alliance is positively linked to clients’ perceptions of therapist empathy and sincerity (‘genuineness’). This indicates that these relational aspects are closely connected in the subjective experience of beneficiaries (Nienhuis et al., 2018). The practitioners’ personal qualities, especially their deep understanding of both the professional mental health context and the lived experience of caregiving, proved central to the positive relational experiences described by informal caregivers. Specifically, flexibility, adaptability, and co-creation allowed for ongoing adjustments to session content and pacing to better meet caregivers’ needs, concerns, and emotional states. Practitioners relied on participants’ personal experiences to co-develop reflection opportunities or practical exercises. For some caregivers, this flexibility helped address specific challenges or introduce new tools, enhancing both the relevance of the support and recognition of their experiential knowledge. These findings align with the concept of therapist ‘responsiveness’ (Davì et al., 2024), which refers to the ability to adapt behavior moment by moment to the unique dynamics of the therapeutic relationship. They also support recent research emphasizing practitioners’ capacity to modify session content and pace in real time, tailoring interventions to individual needs and improving engagement (Braun and Clarke, 2006; Esposito et al., 2024). The calm and relaxed atmosphere fostered within the Ensemble program—largely shaped by the practitioner’s stance—emerged as another key facilitating factor reported by caregivers. They emphasized that the combination of attentive listening and a secure, supportive environment enabled them to progress at their own pace, concretely experiment with new coping strategies, and ultimately engage more fully in a process of change. In turn, this ongoing process of change further strengthened their overall involvement in the program. These observations also align with psychoeducational intervention literature, emphasizing that flexibility, individualization, and co-construction enhance the engagement, relevance, and recognition of caregivers’ knowledge (Ponce et al., 2011; Stubbe, 2018; Tessier et al., 2023), as recommended by the Ensemble program (Rexhaj et al., 2017b). Moreover, because the lived experience of informal caregivers supporting individuals with mental health disorders is often poorly understood by healthcare professionals—and even more so by society at large, frequently giving rise to stigmatization—the findings of this study highlight the importance of training professionals on the specific challenges associated with the caregiving role in psychiatric contexts (Kallio et al., 2016). Indeed, the empathic stance adopted by practitioners in the Ensemble program can only be fully effective because it is grounded in a solid understanding of what the caregiving role entails, including the high emotional burden, the unpredictability of symptoms, and the chronic course of many psychiatric conditions. The practitioners’ expertise is all the more crucial in that it enables the validation of informal caregivers’ experiential knowledge, creating relational symmetry aligned with the principles of patient recovery and partnership (Pomey et al., 2015; Slade, 2009).

These two relational dimensions—an empathic, respectful, accepting, and partnership-oriented stance, along with flexibility, adaptation, and co-construction—seem especially important, as they were also spontaneously recognized by most informal caregivers as the most meaningful aspects of the program. Indeed, in response to the open-ended question about what they valued most in the Ensemble follow-up, caregivers consistently emphasized the practitioners’ professionalism, non-judgmental attitude, attentive listening, and the sense of being genuinely supported. They also highlighted the value of having a guided space for personal reflection and structured engagement, which collectively contributed to their positive experience of the intervention. Indeed, neutrality and non-judgment also enable caregivers to acknowledge and embrace caregiving roles (Norcross and Wampold, 2011; Rogers, 1970). Informal caregivers emphasized their deep appreciation for the intervention’s unique space for attentive listening and experiential sharing.

Conversely, the findings of the present study suggest that when the informal caregiver reported only moderate satisfaction with the Ensemble program, the practitioner experienced a weaker sense of relational closeness, likely reflecting a form of reciprocity within the interaction. The practitioner’s account further shows difficulties in maintaining an empathetic stance toward the care recipient, instead taking a more detached approach. Simultaneously, the practitioner’s tendency to structure the support sessions more rigidly seems to indicate less flexibility in responding to the informal caregiver’s expressed needs or expectations.

Regarding the two other aspects of the therapeutic relationship—therapeutic alliance and relational authenticity—our findings suggest that while both are crucial for the intervention’s effectiveness and the overall relationship quality, they were mentioned less spontaneously by informal caregivers when describing their experience. It may simply be less common for intervention recipients to explicitly reflect on their own experience of the therapeutic relationship. The few informal caregiver excerpts that do address these aspects highlight the delicate and permeable boundary between maintaining a professional stance and expressing authenticity, often emphasizing the practitioner’s inherent personal qualities. Such authenticity seems to promote a sense of reciprocity within the dyad. Concerning therapeutic alliance (understood here as a shared agreement on the intervention’s goals), informal caregivers described it as a collaborative process that gradually helped them see the program’s relevance for themselves, especially in connection with their caregiving role. These findings highlight a particular challenge frequently encountered in interventions targeting informal caregivers: negotiating the focus of care. Informal caregivers often experience heightened distress and deteriorating health, yet they tend to prioritize the needs of their ill relative over their own (Rexhaj et al., 2017b). Consequently, informal caregivers commonly enter support programs with expectations focused on obtaining help for their relative rather than for themselves (Rexhaj et al., 2017a). In the present study, informal caregiver interviews reveal that the framework of the Ensemble intervention, namely, that the primary beneficiary is the caregiver rather than the care recipient, had to be clearly established and repeatedly communicated, allowing caregivers to gradually recognize the intervention’s objectives. This finding resonates with Bordin’s (1979) concept of the therapeutic alliance, which emphasizes that therapeutic work can only progress when both parties share a clear and nuanced understanding of the intervention’s goals. Indeed, any misalignment in this understanding may undermine the quality of the therapeutic relationship if not explicitly addressed, as also indicated by practitioner reports in the present study.

A key strength of this study is its design, which combines practitioners’ perspectives with caregivers’ varying satisfaction levels with the intervention. This method provides a unique chance to identify and analyze specific aspects of the therapeutic relationship that could serve as critical points, potentially influencing caregivers’ perceptions of dissatisfaction with both the intervention and, in some cases, the therapeutic relationship itself. These two dimensions of the therapeutic relationship (therapeutic alliance and relational authenticity) appear to have posed the greatest challenges for Practitioner B during the intervention with Participant 3, who reported only moderate satisfaction with the Ensemble program. The practitioner observed a misunderstanding regarding the therapeutic goals on the part of the caregiver, likely reflecting the caregiver’s difficulty in accepting the brief and caregiver-focused nature of the intervention. Additionally, the practitioner reported feeling uncomfortable within this relationship, regretting not having shared or expressed her own experiences at that time with the caregiver. Indeed, not explicitly addressing the therapeutic relationship with the caregiver may weaken the alliance itself. In the literature, the concepts of “processing the relationship” (Hill et al., 2018; Hill and Knox, 2009) and “immediacy” (Iwakabe and Conceicao, 2016; Rexhaj et al., 2020; Shafran et al., 2017) refer to the practice of directly and promptly discussing the relationship by inquiring about, or disclosing, one’s immediate feelings. In Ensemble intervention, this may involve the practitioner taking a moment at the end of each session to comment on their feelings toward the informal caregiver, their feelings about themselves in relation to the informal caregiver, or perceptions of the therapeutic relationship more broadly. Indeed, openly discussing the relationship may help prevent or reduce the likelihood that relational difficulties—when perceived as troubling by either the therapist or the client—persist beyond the session or even lead the client to prematurely terminate therapy. However, discussing the therapeutic relationship when difficulties arise can be challenging. Some authors even suggest that addressing problems within the therapeutic relationship may draw excessive attention to what is not working, appear intrusive or inappropriate, and ultimately exacerbate the issue (Horvath and Bedi, 2002; Safran and Kraus, 2014). At the same time, failing to acknowledge such disruptions can be equally detrimental to the relationship itself (Zlotnick et al., 2020). The therapist, or in this context, the practitioner, therefore faces a genuine dilemma: addressing relational difficulties in an attempt to repair them, while also risking further strain on the relationship. In any case, although the evidence remains limited, some studies suggest that discussing the relationship when it is functioning well may be particularly beneficial, capitalizing on positive dynamics to strengthen the therapeutic alliance and foster engagement in treatment (Solomon and Berman, 2025).

Overall, all the results of this study showed that although practitioners maintain professional boundaries, sometimes at the risk of losing authenticity, informal caregivers value their personal qualities (such as empathy and respect) and ongoing adaptation to the caregiver’s experience and circumstances across the five Ensemble sessions. Attentive, accurate, and non-judgmental listening fosters informal caregiver engagement and promotes active participation in co-constructing interventions. These interactions reflect a synchrony between Ensemble practitioners and informal caregivers, emphasizing the interdependent and dialogical nature of the therapeutic relationship. Practitioners respond to what caregivers share; caregivers, in turn, integrate what practitioners offer, maintaining a flexible, reciprocal balance of giving and receiving. This sense of symmetry in the relationship enables genuine encounters between practitioners and informal caregivers. The caregivers feel free to share their experiences and perspectives when they perceive that their values and viewpoints are acknowledged, while also benefiting from the practitioner’s expertise in the challenges of psychiatric caregiving. In this study, the specific case of the practitioner’s experience of the therapeutic relationship with the informal caregiver—who reported moderate satisfaction with the Ensemble intervention—also highlights the crucial and challenging role of the first Ensemble session in establishing a solid therapeutic relationship. During the first meeting, the practitioner must strike a balance between several skills: defining the scope of the intervention, clarifying the objectives, and adopting a welcoming and non-judgmental attitude toward the caregiver’s unique experience. These elements are essential for fostering a complementarity between experiential and professional knowledge. Such complementarity promotes early engagement in the change process by making the intervention goals feel attainable and by strengthening the informal caregiver’s sense of being supported. It also facilitates greater flexibility and the co-construction of the therapeutic relationship. As informal caregivers commit to change, they become more open to experimenting with new situations or exercises the practitioner proposes, who then adapts to the informal caregiver’s context. Beyond a cognitive understanding of their experiences, informal caregivers were able to live through the process across the five sessions. This shared approach strengthens reciprocity within the relationship and allows the practitioner to move away from a results-driven mindset or the pressure of a brief intervention. A virtuous dynamic emerges, where the balance between professional stance and relational symmetry enables full, humanized engagement. This represents a unique strength of a brief, non-standardized, person-centered intervention such as the Ensemble program.

## 5 Limitations and future research directions

The results of this study should be interpreted carefully and confirmed through future research, as several methodological limitations need to be acknowledged. In particular, the participant sample was relatively small, which emphasizes the exploratory nature of the study. Nevertheless, analyzing the therapeutic relationship among caregivers who were more or less satisfied with the Ensemble intervention offers an original contribution and allows for discussing situations where the therapeutic relationship presents specific challenges. Additionally, even though the number of participants was limited, the study combined data from different sources and diverse qualitative methods (such as individual research interviews with Ensemble practitioners and a focus group with caregivers). This methodological triangulation helped reduce biases inherent to each approach and, importantly, enabled exploring the intersubjective dimensions of the relationship by considering both practitioners’ and caregivers. Few studies in literature have implemented methodological designs that capture this intersubjectivity through the narratives of both parties involved in the relationship (Flückiger et al., 2020; Sandhu et al., 2015), as proposed here. To our knowledge, no study to date has examined the intersubjective dynamics between caregivers of individuals living with severe mental disorders and healthcare professionals. Nonetheless, the present study did not allow for a direct analysis of the interactional processes within the therapeutic relationship. Such an analysis would involve, for example, video-based observation of both actors in the relationship throughout one or multiple sessions, using a dynamic, interaction-focused approach. Another limitation is that caregivers were not explicitly asked about their experience of the therapeutic relationship itself, but rather about their overall experience with the Ensemble intervention. Using a questionnaire specifically designed to assess the therapeutic relationship or its dimensions might have led to different conclusions. Future research should utilize single-case studies to analyze practitioner–caregiver interactions across all program sessions, providing insights into maintaining reciprocity over time. This approach would enable a deep understanding of how relational dynamics and intersubjective processes develop over time, including pinpointing key moments when shared understanding is formed or broken. Additionally, incorporating detailed conversational or sequential analyses (Hill et al., 2018; Hill and Knox, 2009; Iwakabe and Conceicao, 2016; Shafran et al., 2017) of these interactions could offer insights into the micro-processes that maintain intersubjectivity, such as turn-taking, tuning into emotional cues, and the co-construction of meaning.

Beyond the Ensemble program, several findings could apply to other brief interventions involving informal caregivers. Focusing on intersubjectivity, quick goal alignment, and genuine engagement aligns with research on family psychoeducation and recovery-focused practices, which emphasize the importance of collaborative, co-created relational processes (Kaas et al., 2003; Rodolico et al., 2022). These relational mechanisms might therefore be relevant for informal caregiver interventions in various settings, such as early psychosis services or community support programs, where caregivers encounter similar emotional and relational challenges. From a training perspective, these findings also underscore the importance of integrating modules focused on the therapeutic relationship within programs designed for brief interventions. Specifically, this involves developing competencies in relational attunement, rapid alliance formation, and managing goal divergences—dimensions that have been shown to predict effectiveness across other therapeutic modalities (Flückiger et al., 2018; Norcross and Lambert, 2018). Additionally, incorporating structured supervision that explicitly addresses intersubjectivity, countertransference, and the negotiation of relational asymmetry could further enhance practitioners’ capacity to conduct brief interventions in a nuanced and effective manner. Experiential learning also allows practitioners to test and expand their own capacity to engage in the process of change.

Originally, this study examined the therapeutic relationship in a brief intervention for informal caregivers, a group rarely studied outside of patient-centered research. However, the World Health Organization (2022) has recently redefined informal caregivers not as peripheral support sources but as active partners in care. Therefore, it is essential to increase empirical research and the evaluation of programs directly aimed at caregivers of individuals with mental health disorders. Advancing this research should, in turn, help train healthcare professionals by increasing awareness of the challenges caregivers face. Such progress may foster attitudes and practices that value caregivers’ experiential knowledge and, as a result, improve their involvement in support services. Without these developments, informal caregivers risk becoming the next patients themselves.

## 6 Conclusion

In conclusion, the therapeutic relationship between Ensemble practitioners and informal caregivers is mainly based on the quality of shared experiential processes, which shape key relational aspects like alliance and empathic, respectful collaboration. The current findings emphasize reciprocity as a key mechanism of the relational experience, supporting both recognition of informal caregivers’ needs and the maintenance of a reflective professional stance among practitioners. These results also suggest that developing effective relational dynamics quickly in brief interventions depends on skills that promote intersubjectivity and co-construction processes. Ultimately, reciprocity stands out as a crucial concept that warrants further empirical research, especially regarding its impact on both informal caregivers and more generally care recipients.

## Data Availability

The original contributions presented in the study are included in the article/Supplementary material, further inquiries can be directed to the corresponding author.
