# Supplementary material for: Fostering therapeutic relationships in brief interventions: an exploratory qualitative study of the Ensemble program for informal caregivers of adults with psychiatric disorders
Source: Front Psychol. 2026 Mar 5;16:1706122. doi: 10.3389/fpsyg.2025.1706122 (PMC12999909; doi:10.3389/fpsyg.2025.1706122)
Supplement: Supplementary file 1 [file Supplementary_file_1.docx]

Supplementary Material 1

# **Semi-Structured Interview Guide**

Practitioners – *Ensemble* Program

**Opening Questions on the Therapeutic Relationship**

**Primary question**

- How did you experience the relationship between yourself and the informal caregiver you followed throughout the Ensemble program? Could you describe this relationship?

**Probing questions**

- What were your expectations?
- What were your apprehensions?
- What difficulties did you encounter?
- What were the positive aspects?

**Experience of the Therapeutic Relationship**

- In your view, what were the most important and most salient elements of the relationship between yourself and the informal caregiver?
- Are there any particular elements that come spontaneously to mind when considering this relationship and its importance?

**Therapeutic Alliance**

- How would you describe the quality of the therapeutic alliance with the informal caregiver?
- What strategies did you use to develop and maintain this alliance?

**Practitioner Characteristics and Attitudes**

- What attitude and professional stance did you choose to adopt toward the informal caregiver you supported?
- Did you notice any discrepancy between the stance you initially intended to adopt and what emerged spontaneously during the interviews?

**Empathy**

- What emotional reactions did you experience toward the informal caregiver?
- Did you experience empathy toward the informal caregiver you supported?
- How would you characterize or describe this empathy?
- Were you able to rely on this empathy during subsequent exchanges? If so, in what way? (Examples?)

**Unconditional Positive Regard and Non-Judgment**

- How did you welcome the informal caregiver and their way of being?
- How did you respond to their attitudes, values, personality, and judgments?
- Do you think you were able to adopt an attitude of unconditional acceptance and non-judgment toward the informal caregiver throughout the follow-up?
- (If yes or no) How do you think this influenced the relationship?

**Authenticity (Congruence)**

- How would you describe your attitude toward your own emotional experiences within the relationship, your way of observing them, and, where appropriate, communicating them?
- Do you think you were authentic (congruent) in this relationship?
- How would you describe this attitude of authenticity?
- Were you able to rely on this authenticity during the exchanges? If so, how? (Examples?)

**Practitioner’s Capacity to Manage Relational Ruptures**

- Were there situations you experienced as difficult during the Ensemble sessions?
- Did you perceive moments of tension or rupture in the relationship? (Examples?)
- If so, how did you experience and manage these situations?
- Was the situation resolved from your perspective?
- If so, what strategies and attitudes did you implement to achieve this positive development?

**Capacity to Provide a Secure Base and to Manage Emotional Engagement**

- Did you identify a specific attachment style in the informal caregiver (if applicable), and did you take this into account in your support?
- Did you implement specific elements to provide a secure base within the therapeutic relationship? Which ones? How?
- How did you approach separation and the end of the intervention? What elements did you put in place to facilitate this separation?

**Adaptation to the Person and Sensitivity to Individual Characteristics**

- How would you describe your methods and interventions, particularly in terms of flexibility? What strategies did you put in place to adapt to the specific characteristics of the person you supported?

**Closing Question**

- Is there any aspect of your relationship with the informal caregiver that has not yet been addressed and that you would like to add before we conclude this interview?
