# Supplementary material for: Fostering therapeutic relationships in brief interventions: an exploratory qualitative study of the Ensemble program for informal caregivers of adults with psychiatric disorders
Source: Front Psychol. 2026 Mar 5;16:1706122. doi: 10.3389/fpsyg.2025.1706122 (PMC12999909; doi:10.3389/fpsyg.2025.1706122)
Supplement: Supplementary file 2 [file Supplementary_file_2.docx]

**Supplementary Material 2**

**Focus Group Interview Guide**

*French Pilot Study-* *Ensemble Program*

| **Experience of Informal Caregivers Regarding the *Ensemble* Program** | |
| --- | --- |
| **Introduction**  **Welcome and Facilitator Introduction**  Thank you very much for coming back to meet with us today.  Thank you for agreeing to participate in what we call, in our professional terminology, a focus group.  This simply consists of bringing together people who have benefited from the same program. The idea is to enable them to exchange in a group about the program they participated in and about their feelings regarding their participation in this program.  Once again, we thank you for the time you are devoting to the completion of this project  My name is [First name, Last name]. I am acting here as a research assistant. I work with Hélène Wilquin, Associate Professor in Psychology at Aix-Marseille University (AMU), Léa Plessis, PhD candidate in Psychology (AMU), and Professors Shyhrete Rexhaj and Jérôme Favrod from the Haute École de Santé Vaud/La Source School of Nursing in Lausanne, Switzerland.  I am very pleased to meet you this morning  **Introduction to the research**  As Léa and Hélène informed you by email, we aim to qualitatively understand the experience of participants in the *Ensemble* program. Today’s focus group will help us to better understand your experience in particular and the reflections that may emerge from your exchanges within this group  **Focus Group Procedure**  The group discussion this morning should last approximately 1.5 to 2 hours at most.  **Confidentiality**  I guarantee that the content of these exchanges will be used solely for scientific research purposes and that your identity will remain confidential. The analyses will focus on the exchanges that take place this morning. There will be no way to identify you personally.  **Audio recording**  If you do not mind, as stated in the consent form, I would like to audio-record this session to facilitate note-taking and to allow me to be fully present in our conversation. The recordings will not be disseminated or shared outside the research team  **Participant Comfort**  Is everything clear and acceptable to you? Do you have any questions?  Opening Question of the Focus Group   - **Could you describe your experience of the *Ensemble* program?** | |
| **Objective:** To understand participants’ experiences of the *Ensemble* program | |
| **Questions** | **Objectives** |
| **Participants’ Perception of the Support Received**   - **How would you describe the support you received through the *Ensemble* program?** | - To identify the potential added value of the *Ensemble* program for both the informal caregiver and the care recipient - Identify, if applicable, how the program content has contributed to participants’ ability to manage difficult emotions, to address current or future challenges, and to exercise agency either in their role as an informal caregiver or independently of this role. - Identify potential areas for program improvement. - Explore possible change processes that the program may have initiated, and collect concrete examples illustrating these changes. - Assess participants’ understanding and acceptance of the term “informal caregiver.” Does this term resonate with them? - Attempt to identify key factors that facilitate engagement or lead to disengagement in the program. - Explore, implicitly if necessary, the importance of the participant–practitioner relationship for program success and its perceived effectiveness for the participant. - Gather participants’ suggestions for potential improvements to the program. |
| **Evolution of the Participant’s Situation and Change Processes**   - **Has your situation changed since participating in the *Ensemble* program compared to how it was at the beginning?**  \| YES \|  \| \| --- \| --- \| \| NO \|  \|   If Yes :  In what ways is your situation different?  Do you attribute this change to your participation in the *Ensemble* program?  Which elements of the program do you believe contributed to this change or helped you initiate it? |  |
| **Engagement in the Program**   - **Could you describe a particularly significant situation you experienced during the *Ensemble* program?** - Can you tell us what helped you cope with this situation? - Is there anything you wish had been done differently, or anything you feel was insufficient during this situation |  |
| **Evolution of the Care Recipient’s Situation and Possible Link with the Caregiver’s Participation in the Program**   - **Has your relative’s health status changed since participating in the *Ensemble* program compared to the beginning?**  \| YES \|  \| \| --- \| --- \| \| NO \|  \|   In what ways has your relative’s health status changed?  Do you believe this improvement is related to the support you received through the program?   \| YES \|  \| \| --- \| --- \| \| NO \|  \|   If YES, how would you describe the link between this improvement and your participation in the *Ensemble* program? |  |
| **The Term “Informal Caregiver”**   - **What are your thoughts on the term “informal caregiver”?**   Has the *Ensemble* program influenced the way you relate to or identify with this term, either positively (adopting it) or negatively (rejecting it)?  Please elaborate on your answer |  |
| **Suggestions for Improvement and General Feedback on the *Ensemble* Program**  Do you have any comments or suggestions for improving the support you received through the program? |  |
| **Final thoughts**  If you had to summarize your experience as a participant in this program in one word or phrase, what would it be? |  |
